# Study of Surface Displacements on Tunnelling under Buildings Using 3DEC Numerical Modelling

**DOI:** 10.1155/2014/828792

**Published:** 2014-10-29

**Authors:** Nalini Rebello, V. R. Sastry, R. Shivashankar

**Affiliations:** ^1^Department of Civil Engineering, National Institute of Technology Karnataka, Surathkal 575025, India; ^2^Department of Mining Engineering, National Institute of Technology Karnataka, Surathkal 575025, India

## Abstract

Underground structures at shallow depths are often constructed for metro lines, either in loose or dense layered soils. Tunnelling in urban areas is predominantly under surface structures and on tunnelling, innumerable changes in the form of distortion take place in strata surrounding the tunnel. Extent of displacement/damage to buildings or the tunnel-soil structure interaction depends on the type of building and nature of strata. Effect on displacements has been less studied in granular soils compared to other types of soils like clays. In this paper, parametric studies are conducted to find the displacements at surface, in granular soil conditions, due to varying building storeys and building eccentricities from the tunnel centre line. Effect of presence of geosynthetic layer under footings is further studied. Prior to the parametric studies, validity of the model used is checked with field data available for a stretch of tunnel in South India. Results of simulation studies reveal that inclusion of building reduces displacements at the surface in the dense strata. In very dense strata, the displacements increase as compared to the case without a building. As the centre of the building moves away from the tunnel centre line, settlement above the tunnel matches displacements in the case without building. Applicability of 3DEC software is checked with respect to the present study.

## 1. Introduction

Underground openings like tunnels for roads, metros, and sewage transportation are constructed in most parts of the world. The depth of the tunnel depends on various factors such as the purpose of the tunnel constructed, alignment, and geological considerations. Shallow tunnels like metro tunnels are constructed in softer strata and under preexisting buildings. When tunnelling operations are carried out at shallow depths overburden displacements generated at the crown are transferred as displacements to the surface and displacement profile generated is in the form of a Gaussian curve [1]. The magnitude of displacements transferred to the surface depends predominantly on the nature of strata between the tunnel and surface. Mair et al. [2] suggest that in soft ground conditions, volume of surface settlements is equal to the volume of ground loss at the tunnel. But in drained condition, the volume of surface settlements is less than settlements at subsurface. However, dense sands show a greater magnitude of movements at *H* > 3*D*, where *H* is the depth of tunnel from surface, as compared to surface movements. Field studies by Attewell and Farmer [3] in stiff overconsolidated clay/London clay indicate that yield of clay at the tunnel face generates 50% of the surface settlement. They attribute to 1/5th of the total settlement to radial yield. Studies by Nunes and Meguid [4] on tunnelling in layered soil, to predict changes in bending stresses in the tunnel lining, installed in soft clay and overlain by coarse grained sand layer, show that increasing the thickness of sand layer and reducing the distance between the coarse grained layer and crown lead to lesser magnitude of displacements.

In the past few years, most researchers have concentrated their studies on ground movements due to tunnelling, in clay soils. Few studies have focused on behavior of tunnelling-induced, ground movements in granular soils. However, detailed experimental investigation by Lee [5], to predict subsurface settlements at shallow and deep depths in granular soils, were found to be in good agreement with results of finite element modeling. Transparent soil models which simulate behavior of granular soils have been also used to investigate subsurface displacement troughs [6, 7]. The measured data is consistent with field measurements and suggests that trough width parameter is independent of the volume loss and is linearly proportional to tunnel depth.

Another factor governing the nature of displacements is the existence of buildings. Previous research, in the presence of buildings, has been conducted using numerical modeling investigations (Potts and Addenbrooke [8] and Franzius [9]). Their analysis indicates that the existence of building might alter the displacement profile and hence reduce displacements. Modelling studies conducted under masonry buildings by Liu et al. [10] indicate that the structure follows the profile of the curve generated under Greenfield conditions. Mroueh and Shahrour [11] conducted studies on a symmetrically placed, two-storied framed structure, to predict changes due to excavation-induced ground movements. Overall reduction in displacements, across the transverse settlement profile and longitudinal profile was observed. Furthermore, studies on the effect of multifaced tunnelling in water bearing soft ground by Yoo and Kim [12] revealed that the presence of a structure tends to reduce surface settlement by 15–25% compared to green field conditions. This is because a stiff structure makes surrounding soil stiff and thus reduces settlements.

Previous research on tunnelling has been limited to modeling of a tunnel in the presence or absence of the building, under mixed soil conditions or under clayey soils. Building load was taken as uniform pressure acting at the location of the building and not as loads acting at specific locations, that is, isolated columns/individual columns. Further the effect of geosynthetic layer under a building which is known to act as a base isolator under dynamic loads and as stiffener under static loads was not investigated in the prediction of displacements on tunnelling. Studies on the effect of varying column loads, in varying granular strata conditions and in the presence of a geosynthetic layer, need to be investigated and analysed in order to make in advance predictions of the displacement profile in general and displacements under footings in particular.

Advance prediction of displacements, through numerical modeling investigations, serves as a guide for construction personnel to effectively monitor the progress of a tunnel. Selection of the right constitutive law, which is one of the key parameters in numerical modeling studies, has a significant effect in predicting displacements. Kasper and Meschke [13] used an elastoplastic model, called Cam-clay model, for parametric studies concerning the behavior of ground due to tunneling. Effect of overburden depth, over consolidation, rate of hydration was taken in the analysis. Increase in preconsolidation ratios and overburden reduced settlements; also, the faster the rate of hydration of cement was, the fewer displacements was observed, at surface. Other researchers have made a comparative study of displacements, by taking into account more than one constitutive law. Hejazi et al. [14] carried out studies by using three constitutive laws. They used MC (Mohr-Coloumb criteria), HS model (also called the high stiffness model), and HS small (High stiffness at small strains) to predict settlements of shallow tunnel in overconsolidated clays. Of all the three models, the settlements produced by MC model were wide. Increase in depth of cover increased the settlements in MC model, whereas in HS and HS small model the settlements reduced with depth, which matches with the concept of arching at increased depths form surface. Mašín [15] performed 3D FEM studies on the Heathrow Express trial tunnel in stiff clays with high Ko conditions using two different constitutive laws, the Hypoplastic model and Modified Cam-Clay (MCC) model. Ko value taken at the tunnel level was about 1.5 which is quite realistic for London clay. To study the time dependent stress strain behavior of shortcrete lining a linear elastic perfectly plastic von Mises model with time dependent characteristics were adopted. Results of their studies indicate that the MCC model was not capable of predicting the behavior in the small-strain range and give unrealistic predictions with a surface heave at the top. The hypoplastic model gives more realistic results with a U-shaped surface settlement profile. Most researchers have concentrated their studies on predicting the changes due to excavation by considering the soil mass to follow the Mohr-Coloumb Failure criterion. Nunes and Meguid [4] used Mohr-Coloumb failure criterion to predict changes in surrounding soil mass in soft soils overlain by stiff layer. Lee [5] conducted experimental investigations validated with FEM studies using the Mohr-Coloumb failure criterion for granular soil mass. These experimental investigations matched well with the numerical predictions using Mohr-Coloumb failure criterion and hence in the present case; the stratum is assumed to follow the Mohr-coloumb elastoplastic criterion.

Displacement predictions are commonly carried out by FEM softwares and analysis using discrete elements are hardly known. Hence this paper involves a Three-Dimensional Distinct element study of a tunnel under a building in two different states of granular soils. Three-Dimensional Distinct Element code (3DEC) which is a command driven, Discrete element code is chosen for the analysis, the advantage being material properties like normal stiffness, shear stiffness and coefficient of friction at interfaces can be clearly defined. Although it has been observed that Distinct element codes like UDEC, 3DEC are more suitable for deep underground excavations in rock masses and denser stratum, the applicability of the three-dimensional code for dense and very dense granular stratum has been analysed in this paper.

The focus of this study is to estimate displacement due to tunneling, in coarse grained soils of varying density subjected to varied building loads and eccentricities from the centre line. In order to predict changes in the form of displacements, the study involves three stages. The initial part of the 3DEC modeling involves verifying the numerical modeling procedure adapted, with field data predictions of a tunnel in Bangalore. The second part involves excavation of a tunnel in the absence of a building and the last part of the study involves excavation of a tunnel in presence of the building with varying strata conditions, building storeys, and eccentricities from the centre line. Results of the analysis reveal that in less dense strata with an increase in building load the displacements reduced and in dense strata with an increase in building load the displacements exceeded the displacements under Greenfield conditions.

## 2. Problem Definition and Details about Numerical Modelling

Three-Dimensional Distinct Element code is utilized to study the effect of varying building stories, varied centerline eccentricities on tunnelling induced settlement in granular soils of varying denseness. Prior to the simulation studies numerical model for a stretch of 200 m was developed and results were verified with field instrumentation.

The material model considered for the strata was an elastoplastic model with Mohr-Coloumb failure criterion. A linear-elastic constitutive model was assigned for the tunnel liner and building. To reduce the effect of artificial boundaries, a distance of 8*D* was provided (where “*D*” is the diameter of tunnel) at the sides in the transverse direction and a distance of 17 m was provided from tunnel bottom to the bottom of the model. The entire domain was divided into deformable blocks with each block further discretized into tetrahedrons with predominant geometrical/geological features forming boundaries of different blocks. The average length of each element was 1.5 m. Finer mesh refinement was provided for the building with an average element size of 0.5 m. In distinct element codes like 3DEC, it is necessary to bring the model to a state of equilibrium and therefore unbalanced forces are reduced by increasing the number of cycles or by making changes in the model properties, in the absence of the tunnel. For the parametric studies, the tunnel was excavated in the absence of the building and the change in displacements was noted. Later on, the presence of the building with varying storeys and varied centre line distances was modeled and subsequently vertical and horizontal displacements were measured.

### 2.1. Validity of Model

**Figure 1:**
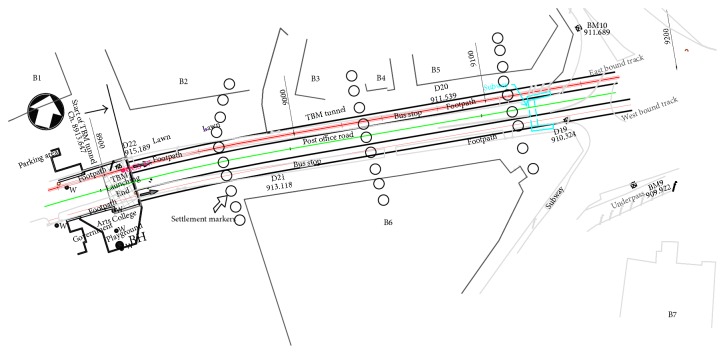
Plan of the tunnel displaying settlement markers.

**Figure 2:**
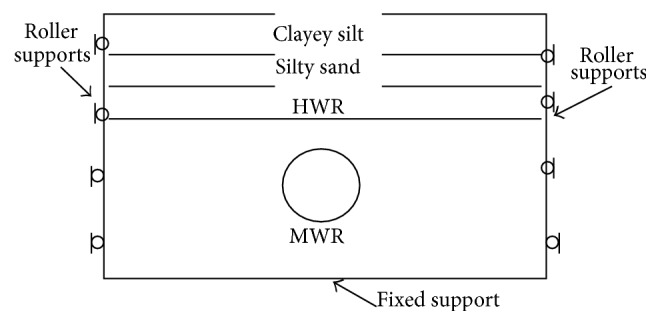
Details of strata and the model considered.

To check the validity of parametric studies, displacements generated by advance of tunnels with building loads applied on either side of tunnels were taken up for analysis. The tunnel under consideration is located in Bangalore and details of the tunnel lining are illustrated in Table 1. Details of the building, settlement markers, and properties of strata are illustrated in Figures 1 and 2 and Table 2, respectively. Borehole data indicated that the materials encountered at the site consisted of 0.5 m layer of clayey silt followed by 0.5 m of silty sand and the subsequent 1 m after the top two layers was made up of highly weathered rock. From a depth of 2 m from surface, strata were comprised of moderately weathered rock with a RMR value of 55 extending to a great depth below. Building weights are applied on the model at B1, B2, B3, B4, B5, B6, and B7, respectively, with a uniform load of −62.5 kN/m^2^, −50 kN/m^2^, −62.5 kN/m^2^, −40 kN/m^2^ and −40 kN/m^2^, respectively, over an area of 21 × 40 sqm, 15 × 20 sqm, 15 × 10 sqm, 20 × 30 sqm, and 80 × 150 sqm, respectively. Volume loss of less than 0.5% is observed during the progress of tunnel. Diameter of the tunnel was 6.1 m with lining thickness of 0.25 m. A constant depth of overburden of 8 m from the surface was taken into consideration.

### 2.2. Details of Simulation/Parametric Study

The numerical model, material models, and details assigned to the tunnel are given in Section 2. Although the site under investigation was predominantly silty sand and weathered rock, in this parametric study changes were only incorporated in the type/denseness of strata and structure of building. A parametric study was carried out involving various geometrical and geotechnical variables in single layer of coarse grained soil of moderate density and in double layers of strata with varying densities of the layers to study the following:

- effect of building storey with varying eccentricity or distances from the tunnel centre line,
- effect of varying building storey on displacements.

#### 2.2.1. Details of the Building

**Figure 3:**
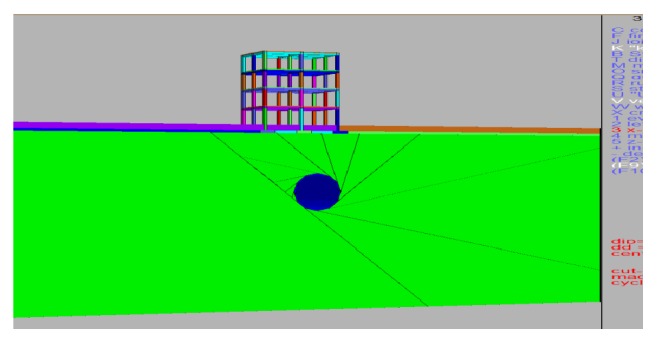
Elevation of building with tunnel.

A framed building without brick-infill walls was considered for the analysis (Figure 3). The centre line of the building varied with respect to the centre line of the tunnel in the transverse direction. The centre line distance of the building varied at *e* = 0 m, 5 m, 10 m, 15 m, and 20 m. Number of storeys of the building varied from 2 storey to 4 storey and 8 storey. Columns are of size 0.35 m × 0.45 m with an axial stiffness of 128 MN. Slab is assigned a thickness of 0.15 m. Beams have cross-sectional dimension of 0.3 × 0.35 m with axial stiffness of 85.8 MN and bending stiffness 0.876 MN-m^2^. Even though the above-mentioned dimensions are characteristics of structures with greater number of floors, slightly oversized beams and columns are provided to facilitate ease in modeling. The footings are of dimensions 2 m × 2 m with a thickness of 0.5 m. To further stiffen the strata under footings, a sand layer sandwiched between two geosynthetic layers of 10 cm thickness was provided under each footing and an angle of friction of 20° was assigned between the soil and geosynthetic layer. A distance of 4 m was assigned from the centre line of one footing to the other, both in the transverse as well as in the longitudinal direction. Bearing pressure exerted by each footing was approximately 24.6 kN/m^2^, 49 kN/m^2^, and 98.549 kN/m^2^ for 2, 4, and 8 storey buildings, respectively.

#### 2.2.2. Details of Simulation Studies in Different Layers of Strata

To analyze the effect of varying strata conditions on tunnelling, two different strata conditions have been analysed.

*(1) Tunnelling in Single Layer of Strata*. In the first case a single layer of strata was taken into consideration. The material was granular soil of uniform density 2000 kg/m^3^ and zero cohesion. Ko value of 0.5 is taken throughout the analysis. The changes in displacements due to excavation, followed by installation of tunnel lining, were noted. Displacements were compared to those under* in situ* conditions of strata/soil mass. The properties assigned to the strata are given in Table 3. 

*(2) Tunnelling in Double Layer of Strata*. In Stage 2 two layers of strata were taken into consideration. Uniform strata were provided up to a depth of 7 m from the surface. From a depth of 7 m a strata having different geotechnical properties of dense strata were incorporated in the modeling studies (Table 4). The changes in displacements due to excavation of the tunnel and subsequent installation of lining were noted.

Changes at the surface and crown were noted for varied building storeys, 2, 4, and 8, at different eccentricities of 0 m, 5 m, 10 m, 15 m, and 20 m, from the tunnel centre line, for all three types of strata.

## 3. Analysis of Results

Results are presented in three stages. In the first stage, validity of numerical model is checked with field investigations based on tunnelling in stages. In second and third stages, change in vertical displacements and horizontal displacements on tunnelling, in single layer of strata and double layer of strata, are studied and presented.

### 3.1. Validity of Modeling

**Figure 4:**
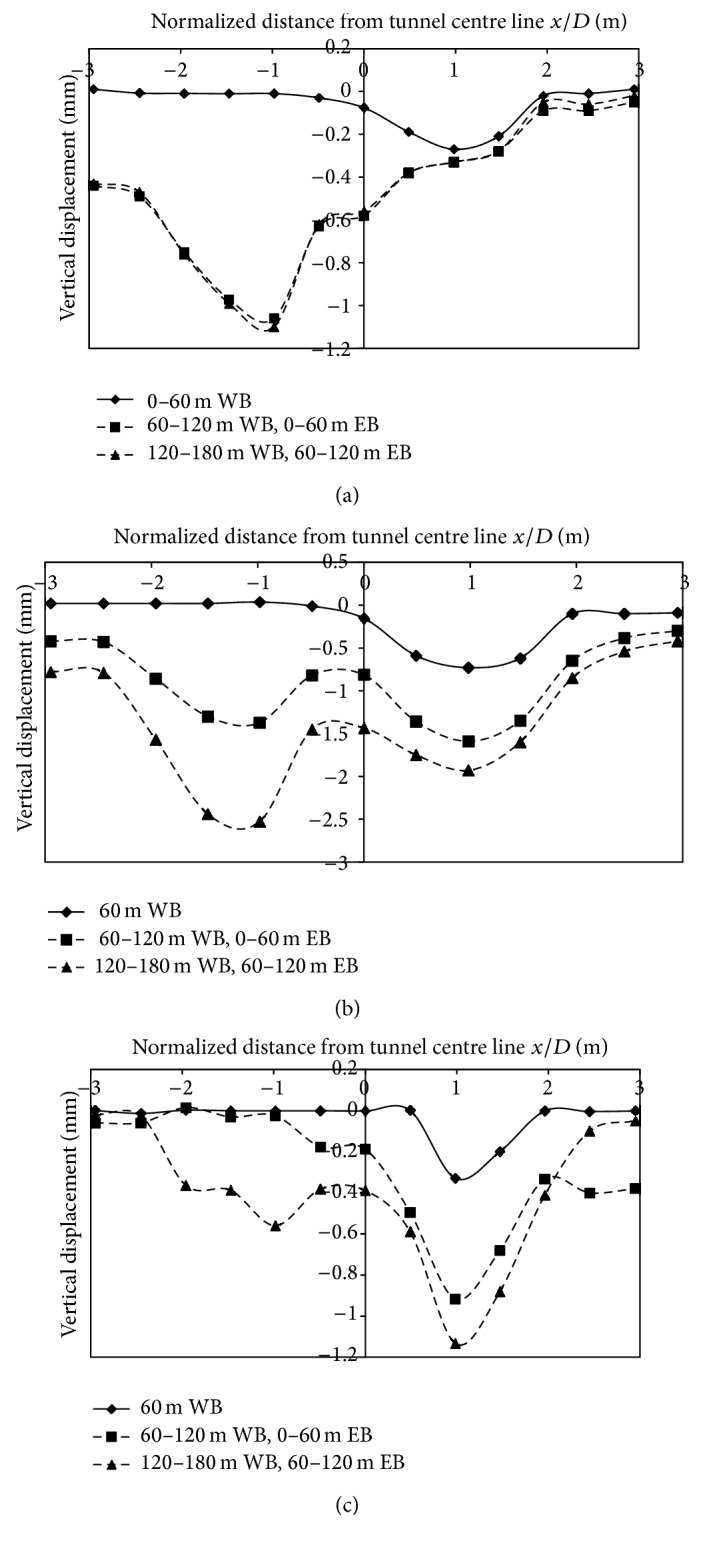
(a), (b), (c): Displacement data generated on numerical modeling at different stages of face progress at Ch. 30 m, 70 m, and 110 m.

**Figure 5:**
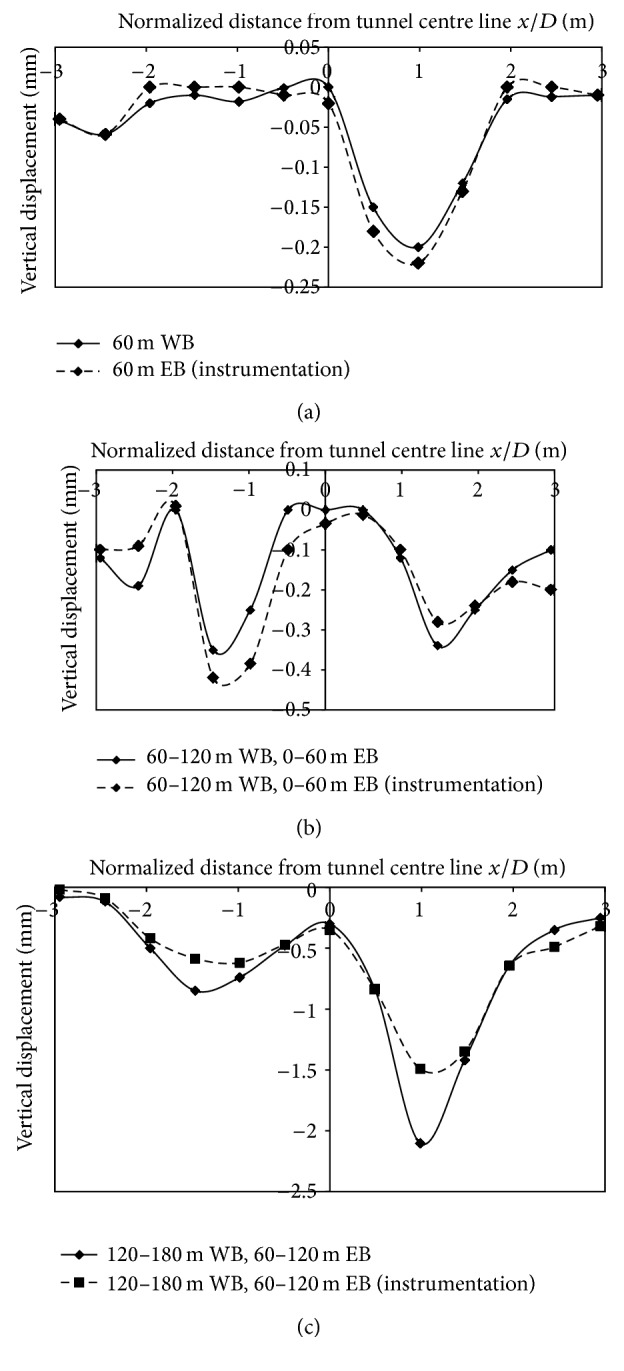
(a), (b), (c): Comparison of numerical modeling with field instrumentation at Ch. 150 m.

Vertical displacements generated at Ch. 30 m, 70 m, 110 m, and 150 m due to stagewise progress of the tunnel, from 0 to 170 m are illustrated, from Figures 4 and 5. The first 0–60 m of excavation of West Bound, tunnel is in purely Greenfield conditions, in the absence of building load. At Chainages 70 m, 110 m, and 150 m, buildings are located on either side of the centerline with the nearest building distance varying from 0–15 m from the centerline of tunnel. Displacements in Figures 4 and 5 are asymmetrical since the simultaneous advance of tunnel face progress is not assessed.

Field monitoring using settlement markers was verified with the result of numerical modeling at Ch. 150 m. Displacements generated during different excavation stages are in good agreement with field instrumentation results and therefore simulation studies could be carried out on the developed model.

### 3.2. Results of Parametric Studies

**Figure 6:**
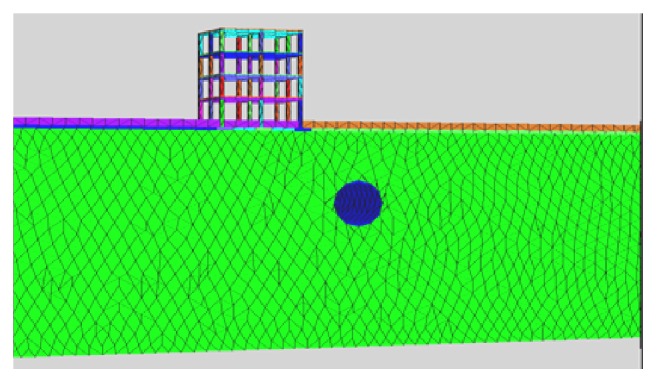
Meshed model with building placed at 10 m from centre line of the tunnel.

**Figure 7:**
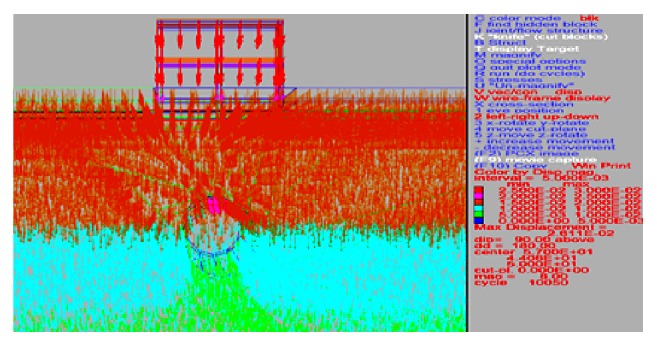
Typical plot of vertical displacements above tunnel.

Results including the mechanism behind the behavior of vertical and horizontal displacements, of the parametric studies, are presented. Model of the tunnel with displacement plot generated is shown in Figures 6 and 7.

#### 3.2.1. Analysis in Single Layer of Strata

Effects of tunnelling in single layer strata were analyzed. The change in vertical displacements with varied building storey and building eccentricity, with respect to the centre line of tunnel, was assessed.

**Figure 8:**
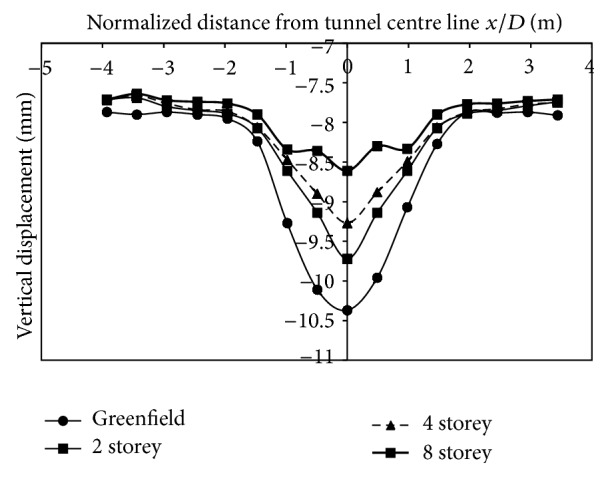
Displacements at surface with building placed on centre line of the tunnel.

*(1) Effect of Varying Building Storey on Vertical Displacements*. The building load taken into consideration was of 2, 4, and 8 storeys. The change in displacements upon excavation, without the presence of building, was −10.37 mm at the tunnel surface (Greenfield conditions) (Figure 8). Inclusion of a building reduced the displacements at the surface as compared to the case without building loads. In the case of a single layer of strata, upon increasing the building load, the displacements reduced. The displacements reduced by 6.26%, 10.6%, and 16.97%, respectively, in 2-storey, 4-storey, and 8-storey buildings, respectively. The main reason for reduction in displacements is that soil surrounding the footing stiffens upon inclusion of building weight and, as a result, overall displacements reduce. With an increase in building load and Ko value of 0.5, greater magnitude of displacements is transferred to the sides of the tunnel and this, therefore, reduces displacements at the crown. The greater the number of storeys is, the greater the transfer of stress to either sides of the tunnel will be. Thus, fewer displacements will be noticed at the crown and surface in less dense granular soils. The reduction in vertical movement is in agreement with studies carried by Mroueh and Shahrour [11], Franzius [9], Potts and Addenbrooke [8], and Yoo and Kim [12]. 

**Figure 9:**
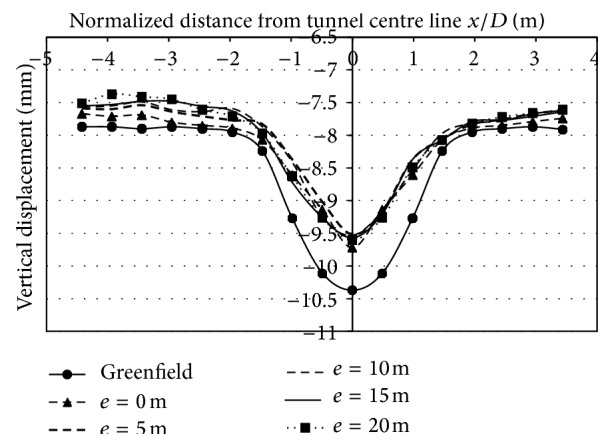
Displacements at surface with 2-storey building placed at varying eccentricities from centre line.

**Figure 10:**
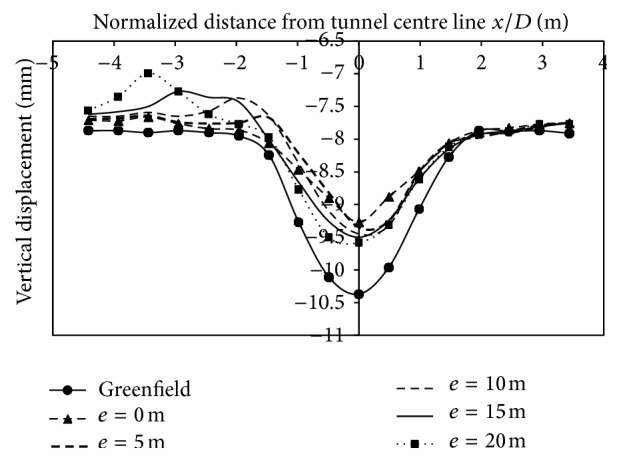
Displacements at surface with 4-storey building placed at varying eccentricities from centre line.

**Figure 11:**
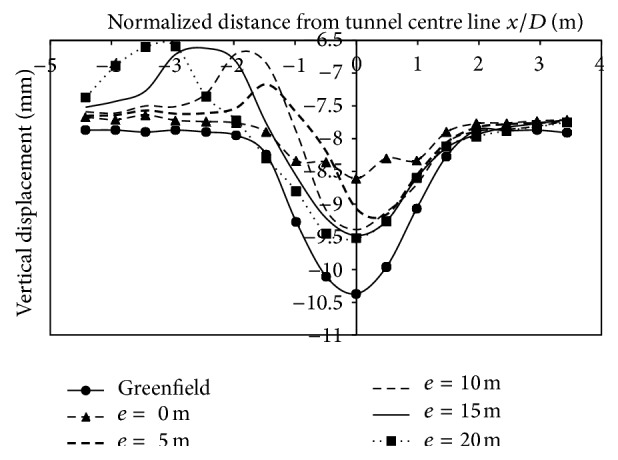
Displacements at surface with 8-storey building placed at varying eccentricities from centre line.

*(2) Effect of Varied Eccentricities on Vertical Displacements*. The effect of varied eccentricities on displacements is studied for all the three building storeys. The eccentricities varied on the left side of the centre line by 0 m, 5 m, 10 m, 15 m, and 20 m. Asymmetric application of load led to asymmetric vertical strata movements at surface. From Figures 9, 10, and 11, it can be observed that the magnitude of displacements was lesser at the location of the footing. The displacements at 3 m (0.49*D*) on the left side of the centerline, when building was placed at 5 m (0.819*D*) from centre line due to 2 storey, 4 storey, and 8 storey, was −9.02 mm, −8.82 mm, and −8.2 mm, respectively. Similarly at −15 m (2.45*D*) from the tunnel centerline, displacements were −7.64 mm, −7.57 mm, and −7.31 mm for 2, 4, and 8 storeys, respectively. As the building eccentricity increased, the displacements at the centre line and on the surface, increased and thus displacements matched the transverse displacement profile created in the case without a building. Even though there is an overall reduction in displacements due to inclusion of building weight, displacements at the footing were of lesser magnitude compared to other points in the transverse direction (Figures 9, 10, and 11; Table 5).

**Figure 12:**
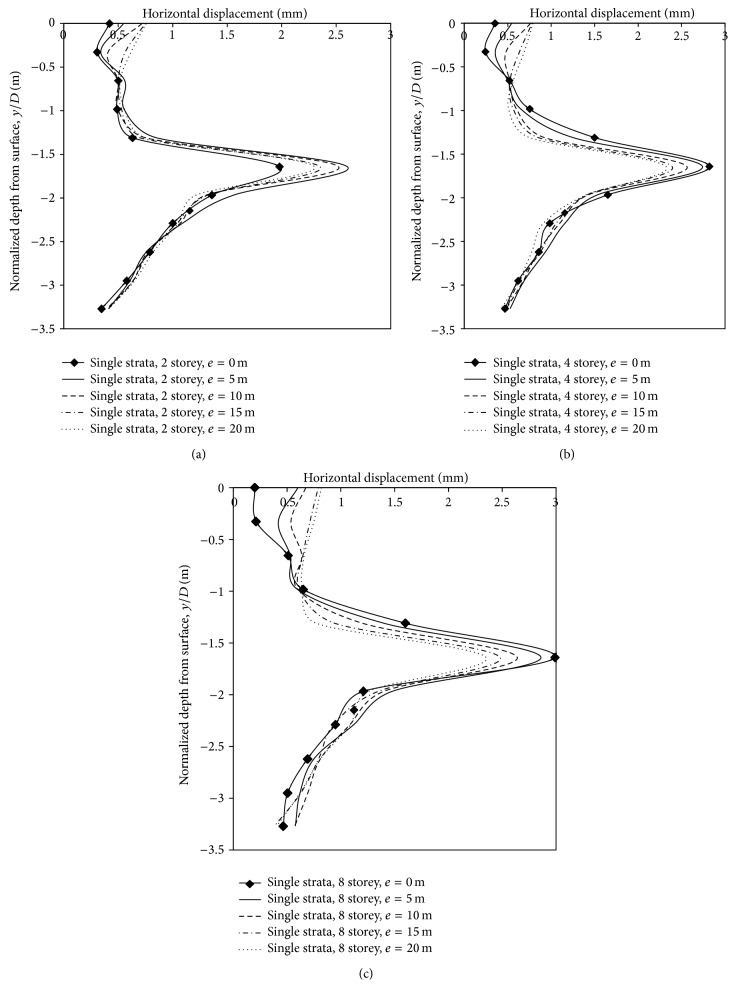
Horizontal displacements along depth with varying building eccentricity and storey.

*(3) Effect of Varying Building Storey and Eccentricity on Horizontal Displacements*. In single layer of strata, horizontal movement in the vicinity of the tunnel increases with an increase in storey. However, for a given storey displacements towards, the tunnel opening reduced as the building moved away from the centre line. Horizontal displacements at an offset distance of 3.1 m on the left side of the centre line (springing level) was 1.98 mm, 2.6 mm, 2.52 mm, 2.35 mm, and 2.3 mm when a two-storey building was placed at 0 m, 5 m (0.819*D*), 10 m (1.63*D*), 15 m (2.45*D*), and 20 m (3.27*D*). Horizontal displacements increased by 42.4% when a 4-storey building was placed on the centre line, compared to displacements generated due to a 2-storey building. Thus, horizontal displacements of 2.82 mm, 2.74 mm, 2.56 mm, 2.39 mm, and 2.32 mm occurred when the building eccentricities were varied from 0 m to 20 m. Placement of an 8-storey building at zero eccentricity resulted in 51% increase in horizontal displacements at the springing level, compared to a 2-storey building. The transfer of displacements to the sides of the tunnel, due to low rise buildings, was less compared to high rise buildings. When the horizontal displacements at the springing level were high, it resulted in lower vertical displacements at the surface. For a given building storey, the reduction in horizontal displacements, as the building moved away from the centre line, is in concurrence with the results of Franzius [9] (Figure 12; Table 6). Hence, high rise buildings in less dense strata reduce the vertical displacements at the crown, and subsequently at the surface.

#### 3.2.2. Effect of Tunneling in Two Layered Strata

In stage two, effects of tunnelling in two layered strata, with tunnel embedded in denser strata and crown at a depth of 1 m, were analyzed. Vertical and horizontal displacements with varied building storey and building eccentricity, with respect to the centre line of tunnel, were assessed.

*(1) Effect of Varying Building Load*. In two layered strata, displacements at tunnel surface reduced upon including building loads. Displacements were of magnitude −3.36 mm before inclusion of building loads and which reduced to −2.5 mm, −2.82 mm, and −3.27 mm, respectively, for the 2-, 4-, and 8-storey building.

**Figure 13:**
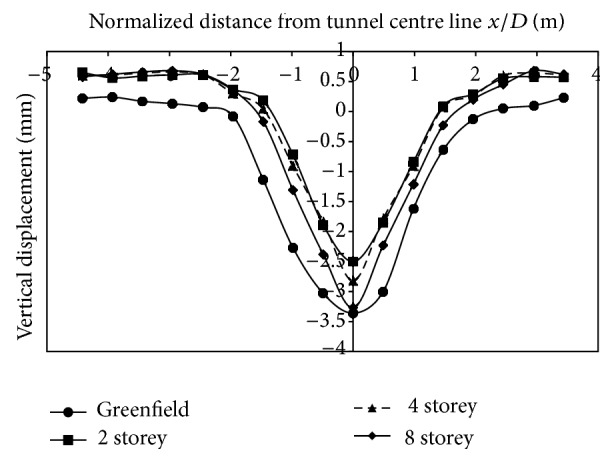
Displacements at surface with building placed on centre line of tunnel.

Thus displacements reduced by 25%, 16%, and 2.67% as compared to the case without a building. Although, there was a reduction in displacements upon inclusion of building loads, displacements increased with an increase in the number of storeys, unlike the case of displacements in single layer of strata where displacement reduced with an increase in building loads. Effect of embedment depth is more pronounced in denser strata. It can be inferred that, in strata with relatively higher density, when the strength of soil almost matches the stiffness of building, the transfer of displacements to the sides of the tunnel are restricted, which leads to greater displacement at the crown than at the sides. Since the zone above the crown is dense, it directly bears the load transferred to it and does not redistribute it to the sides of the tunnel, unlike the case of the tunnel in less dense strata where the soil at the side gets compressed due to load transferred to the sides. Thus, buildings with more storeys, in high strength granular soils, will create more displacements upon excavation of an opening (Figure 13).

**Figure 14:**
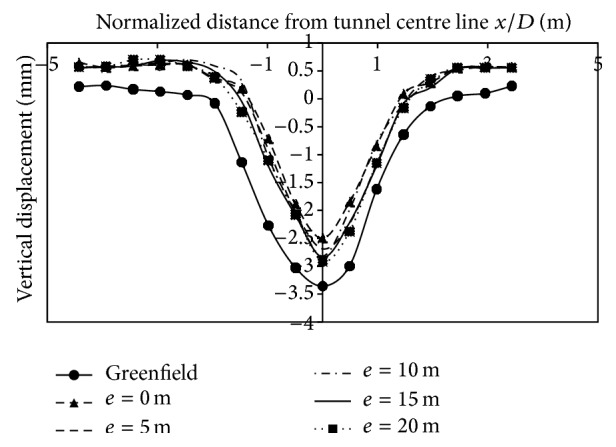
Displacements at surface with 2-storey building placed at varying eccentricities from centre line of tunnel.

**Figure 15:**
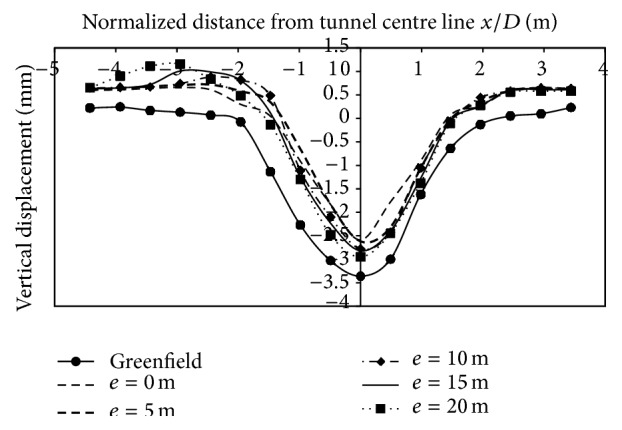
Displacements at surface with 4-storey building placed at varying eccentricities from centre line of tunnel.

**Figure 16:**
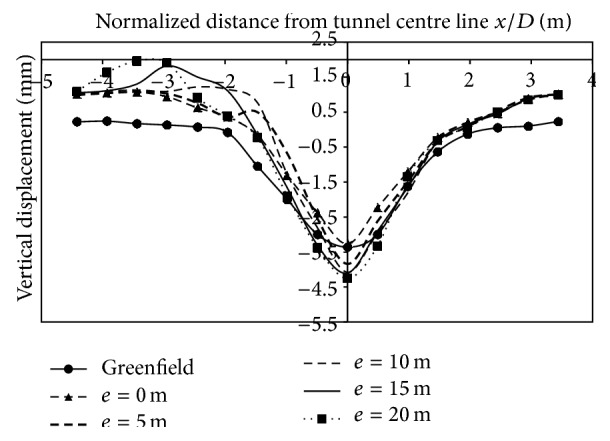
Displacements at surface with 8-storey building placed at varying eccentricities from centre line of tunnel.

*(2) Effect of Varied Eccentricities*. Vertical displacements was of a lesser magnitude as the strata was two layered and the tunnel crown buried under a depth of 1 m of dense strata. Figures 14, 15, and 16, indicate that the magnitude of displacement was lesser at the location of the footing. Displacements at 3 m on the left side of the centre line, when building was placed at 10 m from centre line for 2-storey, 4-storey and 8-storey building, were −2.03 mm, −2.1 mm, and −2.81 mm, respectively. Similarly, at −15 m (2.45*D*) from the tunnel centerline, displacement was −0.61 mm, −0.72 mm, and −0.76 mm for 2, 4, and 8 storeys, respectively. As building eccentricity increased, displacements at the centre line and on the surface, kept on increasing and thus displacements matched the transverse displacement profile created by the case without a building. However, with an increase in building storey (8 storey), the vertical displacements at surface increased and even exceeded the displacements generated without the building load (Figures 14, 15, 16; Table 7).

**Figure 17:**
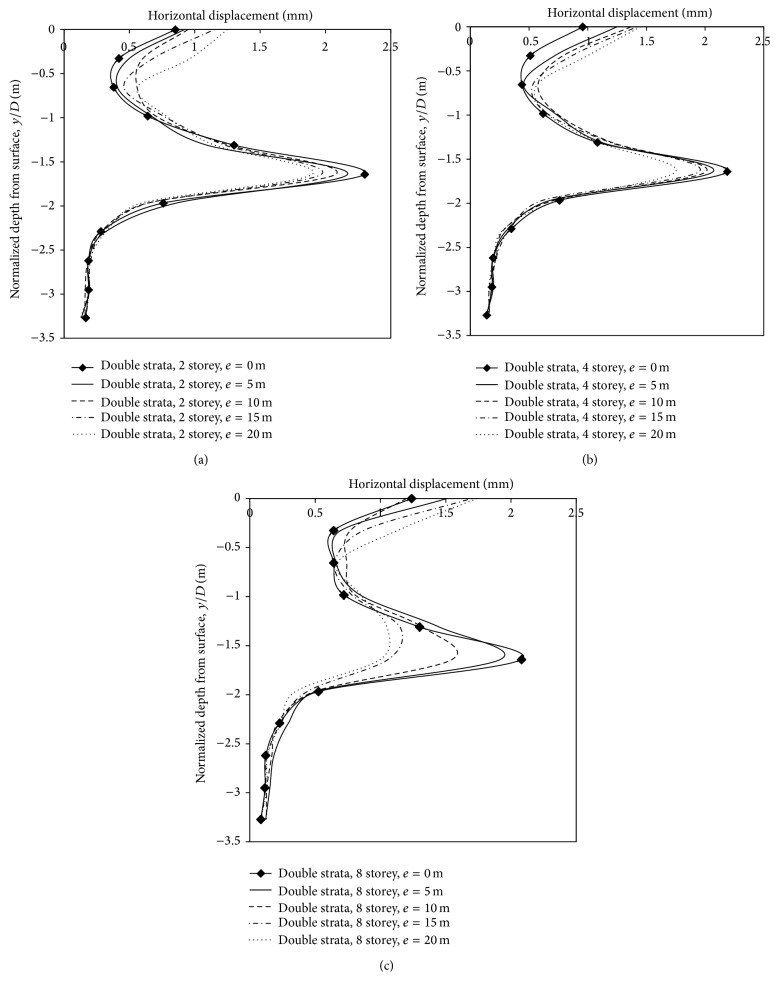
Horizontal displacements along depth with varying eccentricities and storey.

*(3) Effect of Varying Building Loads and Eccentricities on Horizontal Displacements*. In this section, influence of building storeys and eccentricity in two-layered strata was analysed. Horizontal displacements were noticed at 3.1 m on the left side of the centre line (springing level). Displacements were 2.3 mm, 2.19 mm, and 2.08 mm, respectively, when the 2-, 4-, and 8-storey building was placed on the centre line (Figure 17). Interestingly, when the building with varying storeys was placed in less dense strata, horizontal displacements increased with increase in building load, whereas, in a stronger strata, displacements decreased with increase in building storey/load, which can be attributed to lesser soil movement to the sides of the tunnel. In the case of a 2-, 4- and 8-storey building, when the eccentricities of building were varied along the centre line, the horizontal displacements toward the tunnel reduced. Horizontal displacements with a 2-storey building was 2.3 mm, 2.17 mm, 2.08 mm, 1.97 mm, and 1.9 mm, respectively, and with a 4-storey building the displacements was 2.19 mm, 2.07 mm, 2.01 mm, 1.95 mm, and 1.75 mm, respectively, at 0 m, 5 m (0.819*D*), 10 m (1.63*D*), 15 m (2.45*D*), and 20 m (3.27*D*) eccentricities. In the case of an 8-storey building, displacements were 2.08 mm, 1.92 mm, 1.55 mm, 1.06 mm, and 1.02 mm, respectively. Thus, in two layered strata, with tunnel embedded in 1 m of dense strata, reduction of horizontal movements at springing level led to greater strata movements in vertical as well as horizontal direction, at ground surface above tunnel (Table 8).

#### 3.2.3. Analysis of Horizontal Displacements at Surface in Different Types of Strata

**Figure 18:**
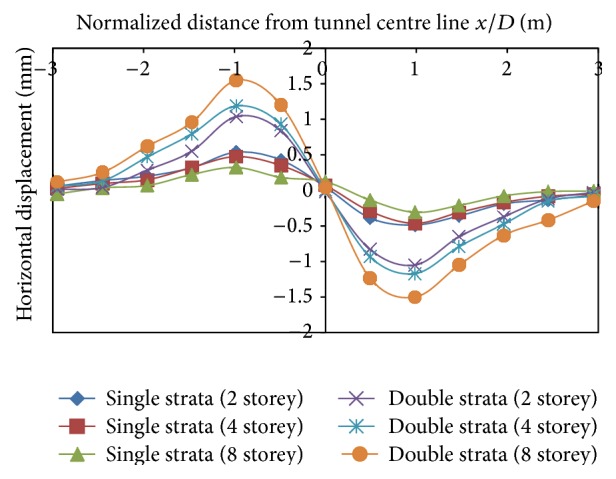
Horizontal displacements on surface of tunnel with varying strata conditions and storey.

The presence of a structure induces more pronounced horizontal displacements at the surface than vertical displacements and hence effect of tunneling on horizontal displacements is studied in this section. In single layer of strata, as the building load increases, the horizontal displacements at surface decrease (Figure 18). At −3 m from centre line, displacements of 0.428 mm, 0.35 mm, and 0.2 mm were noticed, in single layer of strata, with 2 storey, 4 storey and 8 storey. Negative in Figure 18 indicates rightward movement on the left side of tunnel and positive indicates leftward movement on the right side of tunnel. However in two-layer strata, on increasing the building load, the horizontal displacements increased by 0.85 mm, 0.95 mm and, 1.24 mm, respectively, for 2, 4, and 8 storey building loads.

## 4. Limitations of Study

3DEC3.0 is suitable mainly for modeling in stiff clays, rocks, and granular soils of higher density. This study cannot be extended to very loose and soft soils because displacement generated does not actually conform to the Gaussian profile generated due to tunneling in softer soils.

Also, version 3DEC3.0 does not incorporate superior material models compared to 3DEC4.0. This limits the extent to which modeling can be carried out to predict the actual behavior of surrounding soil in softer strata.

## 5. Conclusion

The effect of tunnelling in granular soils of varying densities was studied in this paper. Prior to parametric studies, the model was validated with field observations at a tunnel in Bangalore. Parametric study of the most significant factors, such as building storey and varying eccentricities from the tunnel centre line, on displacement were assessed.

(1) Tunnelling in dense strata, in Greenfield conditions, revealed that displacement profile followed a Gaussian Curve with a maximum displacement of −10.37 mm at the centerline on excavation, whereas, in very dense strata, the maximum displacement was of magnitude −3.36 mm.

(2) Inclusion of a building reduced the displacement as compared to the case without building load, which is in agreement with results by Mroueh and Shahrour [11], Franzius [9], Potts and Addenbrooke [8], and Yoo and Kim [12]. Thus, in less dense soils, increase in building load reduced vertical displacements at surface. It was observed that displacements reduced by 6.26%, 10.6% and 16.97%, respectively, for 2, 4, and 8 storeyed buildings, respectively, when the building was placed on the centre line. In two-layer strata, with varying strata denseness, even though there was a reduction in vertical displacements upon inclusion of building load, it was observed that displacements increased with the increase in number of storeys. This was contrary to the case of displacements in single layer of strata, where displacements reduced with an increase in building load.

Additionally, horizontal displacements increased with increase in building load, in loose strata, whereas, in dense strata, horizontal displacements decreased with increase in building load, which can be attributed to less soil movement to the sides of the tunnel in dense strata.

(3) In relatively loose strata, increase in building eccentricity led to continuous increase in vertical displacements at the centerline, which matched the transverse displacement profile created by the case without a building. Even though there was an overall reduction in displacements due to inclusion of building weight, displacement at the footing was of a lesser magnitude compared to other points in the transverse direction. Similar observations were noted in very dense strata.

(4) The presence of geosynthetic layer in dense strata reduced displacements especially under footing and in very dense strata; the presence of geosynthetic layer led to overall upheaval of displacements at ground surface. Hence, geosynthetic layer can be effectively used in soils which are not very dense. This study can also be extended to tunnels subjected to dynamic loading, with various magnitudes of friction coefficient between the soil and geosynthetic layer.

(5) Three-Dimensional Distinct Element code was found to be effective in analyzing different strata with varying building conditions and eccentricities. It is particularly suited for numerical modeling of strata ranging from dense to very dense soils. Although the studies are conducted on granular soils, the same can be extended to stiff clays and rocks.

Since numerical modelling using 3DEC was in good correlation with field results, the parametric studies involving varying strata and varying building conditions will offer a reliable guideline for engineers in making advance assessment of displacements.

## Figures and Tables

**Table 1:** Properties assigned to the tunnel lining.

Density (kg/m^3^)	Bulk modulus (Pa)	Rigidity modulus (Pa)
2400	1.73*e* ^9^	1.2*e* ^9^

**Table 2:** Rock/soil properties considered for strata.

Layer	Depth from surface	Density (kg/m^3^)	Bulk modulus (Pa)	Rigidity modulus (Pa)	Cohesion (kPa)	Angle of internal friction
Clayey silt	0–0.5 m	1800	1.867 × 10^7^	0.622 × 10^7^	10	32°
Silty sand	0.5 m–1 m	2000	3.9 × 10^7^	2.85 × 10^7^	5	34°
Sandstone/weathered rock	1 m-2 m	2400	3.3 × 10^8^	1.1 × 10^8^	1500	42°
Moderately weathered rock	From 2 m to a great depth	2500	4.76 × 10^8^	1.47 × 10^8^	2000	50°

**Table 3:** Properties assigned to strata.

Density (kg/m^3^)	Bulk modulus (Pa)	Rigidity modulus (Pa)	Angle of internal friction
2000	3.3*e* ^8^	1.1*e* ^8^	32°

**Table 4:** Material properties assigned to different layers of double strata.

Layer	Density (kg/m^3^)	Bulk modulus (Pa)	Rigidity modulus (Pa)	Angle of internal friction
1	2000	3.3*e* ^8^	1.1*e* ^8^	32°
2	2100	8.5*e* ^8^	3.52*e* ^8^	34°

**Table 5:** Vertical displacements on the centre line due to varying building eccentricities.

	gf^*^	*e* ^*^ = 0 m	*e* = 5 m	*e* = 10 m	*e* = 15 m	*e* = 20 m
2 storey	−10.37	−9.72	−9.52	−9.53	−9.57	−9.6
4 storey	−10.37	−9.27	−9.35	−9.45	−9.50	−9.58
8 storey	−10.37	−8.61	−9.07	−9.39	−9.48	−9.52

^*^gf: under Greenfield condition; *e*: eccentricity.

**Table 6:** Horizontal displacements (mm) at the −3.1 m (10 m depth) from centre line due to varying building eccentricities.

	gf^*^	*e* ^*^ = 0 m	*e* = 5 m	*e* = 10 m	*e* = 15 m	*e* = 20 m
2 storey	1.85	1.98	2.6	2.52	2.35	2.3
4 storey	1.85	2.82	2.74	2.56	2.39	2.32
8 storey	1.85	2.99	2.86	2.64	2.48	2.35

^*^gf: under Greenfield condition; *e*: eccentricity.

**Table 7:** Vertical displacements at the centre line due to varying building eccentricities.

	gf^*^	*e* ^*^ = 0 m	*e* = 5 m	*e* = 10 m	*e* = 15 m	*e* = 20 m
2 storey	−3.36	−2.5	−2.69	−2.82	−2.84	−2.91
4 storey	−3.36	−2.62	−2.59	−2.78	−2.82	−2.95
8 storey	−3.36	−3.27	−3.84	−4.08	−4.1	−4.25

^*^gf: under Greenfield condition; *e*: eccentricity.

**Table 8:** Horizontal displacements at the −3.1 m (10 m depth) from centre line due to varying building eccentricities.

	gf^*^	*e* ^*^ = 0 m	*e* = 5 m	*e* = 10 m	*e* = 15 m	*e* = 20 m
2 storey	1.79	2.3	2.17	2.08	1.97	1.9
4 storey	1.79	2.19	2.07	2.01	1.95	1.75
8 storey	1.79	2.08	1.92	1.55	1.06	1.02

^*^gf: under Greenfield condition; *e*: eccentricity.
